# Curriculum initiatives to enhance research skills acquisition by medical students: a scoping review

**DOI:** 10.1186/s12909-021-02754-0

**Published:** 2021-06-02

**Authors:** Crea Carberry, Geoff McCombe, Helen Tobin, Diarmuid Stokes, Jason Last, Gerard Bury, Walter Cullen

**Affiliations:** 1 School of Medicine, University College Dublin, Dublin, Ireland; 2 Health Sciences Library, University College Dublin, Dublin, Ireland

**Keywords:** Medical education, curriculum design, undergraduate research skills

## Abstract

**Background:**

Although it is accepted that providing medical students with opportunities to engage in research activity is beneficial, little data has been collated on how medical degree curricula may address this issue. This review aims to address this knowledge gap by conducting a scoping review examining curriculum initiatives that seek to enhance research experience for medical students.

**Methods:**

This review looks to specifically look at ’doing research’ as defined by the MEDINE 2 consensus rather than ‘using research’ for the bachelor component of the Bologna Cycle. The framework developed by Arksey & O’Malley was utilised and a consultation with stakeholders was incorporated to clarify and enhance the framework.

**Results:**

A total of 120 articles were included in this scoping review; 26 related to intercalated degree options and 94 to non-intercalated degree options. Research initiatives from the United States were most common (53/120 articles). For non-intercalated research options, mandatory and elective research projects predominated. The included studies were heterogeneous in their methodology. The main outcomes reported were student research output, description of curriculum initiative(s) and self-reported research skills acquisition. For intercalated degree options, the three main findings were descriptions of more ‘novel’ intercalated degree options than the traditional BSc, student perspectives on intercalating and the effect of intercalating on medical student performance and careers.

**Conclusions:**

There are several options available to faculty involved in planning medical degree programmes but further research is needed to determine whether research activity should be optional or mandatory. For now, flexibility is probably appropriate depending on a medical school’s resources, curriculum, educational culture and population needs.

## Background

There is a decreasing number of physician scientists at a time when there is an increased demand for evidence based medicine and research [1–3]. Medical schools have a key role to play in this regard, as studies have shown that involving medical students in active research in their undergraduate careers may increase the likelihood that they will be research active after graduation [4–6]. Heparin, insulin, the sinoatrial node and ether anaesthesia are just some of the major discoveries made by medical students [7].

The World Federation for Medical Education (WFME) has listed two standards in relation to medical student research in its 2015 ‘*Global Standards for Quality Improvement in Medical Education’* [8]. The WFME standards incorporate global medical education expert consensus on the best practice minimum requirements (see basic standard definition below) and standards for quality improvement (see quality development standard definition below):

Basic standard: The medical school must throughout the curriculum teach the principles of scientific method, including analytical and critical thinking, medical research methods and evidence-based medicine.

Quality development standard: The medical school should in the curriculum include elements of original or advanced research. Elements of original or advanced research would include obligatory or elective analytic and experimental studies, thereby fostering the ability to participate in the scientific development of medicine as professionals and colleagues. [8]

In 2012, The Association for Medical Education in Europe (AMEE) produced a guide ‘*Developing research skills in medical students’* which recommended that every medical student should understand research methods and the benefits that research brings to their profession [9]. This guide concluded that understanding of research can be greatly enhanced by encouraging the active participation by students in research activities. This correlates with the WFME’s 2015 quality improvement standard. Regulators of medical curricula encourage research. For example, in the UK, in its *Outcomes for graduates* the General Medical Council states under the *Clinical research and scholarship outcome* that “Newly qualified doctors must be able to apply scientific method and approaches to medical research and integrate these with a range of sources of information used to make decisions for care”[10]. Little data has been collated on how medical degree curricula may address this issue. This scoping review looks to address this knowledge gap by specifically examining options to engage students in elements of original research, in terms of ’doing research’ rather than ‘using research’ as discussed by the Thematic Network on Medical Education in Europe (MEDINE2) in *‘Tuning of Research Competencies in Europe’* for the bachelor component of the Bologna Cycle [11].

Previously in 2015, Amgad et al. published an integrated mixed methods systematic review and meta-analysis about medical students’ participation in research [5]. Their main objectives were to examine the short- and long- term influence of curricular and extracurricular undergraduate medical research on the scientific productivity and career choice of medical students.

For this review, we examined both intercalated and non-intercalated degree options because such options are an intrinsic part of some medical degree programmes. Intercalated degree options have been previously reported [12, 13], but not in the context of what is available or undertaken by students who do not complete an intercalated programme. Jones et al’s systematic review covered intercalated bachelor degrees (but not intercalated masters and PhDs). Their main objective was also to measure the effect (in their case, intercalated bachelor’s degrees) on medical student performance and careers.

Active involvement in research may be a positive component of undergraduate medical curricula [5, 12] with benefits that may outweigh negative considerations as described by Simunovic. Simunovic suggested that the majority of medical schools may emphasise student research as an important part of their undergraduate curriculum and that few attempts to elaborate the rationale for such reasoning have been made [14]. Pragmatic considerations of research participation include their impact on medical school applications, their effect on the medical curriculum, their costs, the availability of mentors, and their effects on the school’s educational culture [15]. Cheung questioned the benefits of active research to students and some of their motivations behind doing it [16]. He discussed findings by Pathipati et al. looking at student motivations for doing research [17]. They surveyed students in five medical schools in the US that have highly regarded research programs. Of the 328 respondents, the most common reasons students take years off for research were found to be: “increase competitiveness for residency application” (32 %), “time to pursue other opportunities” (24 %), and “academic interest” (23 %). Issues also discussed by Cheung in his commentary included relatively low publication rates of students in ‘good’ journals and the pressure of academic publishing for medical students from a student’s perspective. The benefits of student medical journals such as the Student BMJ were also discussed but so too was a possible compromise in the quality of articles because of the need to accommodate inexperienced researchers.

Given the potential benefits and disadvantages of medical student research, we carried out this scoping review to map the options available to promote ‘active research’, examine their distribution and outcomes reported.

## Aims

The primary aim was to describe educational activity that involved actively conducting research (‘doing research’) for students on pre-registration medical degree programmes. A secondary aim was to describe the associated benefits and challenges of involving medical students in original research and to explore if there is enough evidence to support an emphasis on medical student research in curricula.

## Methods

A scoping review is defined as ‘a type of research synthesis that aims to map the literature on a particular topic or research area and provide an opportunity to identify key concepts; gaps in the research; and types and sources of evidence to inform practice, policymaking, and research’ [18]. The protocol was designed prior to the scoping review. The framework developed by Arksey & O’Malley (Arksey & O’Malley, 2005) was utilised to conduct a rigorous and methodical search of the current literature. A consultation with stakeholders was then incorporated as advised by Levac [19].

### Stage 1: Identifying the research question

What curriculum initiatives are described in published literature that involve medical students doing original or advanced research?

The principal focus of our research was to examine the curriculum initiatives to enhance original or advanced research skills acquisition on Pre- registration Medical Degree Programmes. For this purpose Amgad and colleagues’ 2015 definition of a medical student was employed, which considers a “medical student” as anyone who is enrolled in the core medical school program, regardless of program duration, and whose graduation would guarantee the degree Bachelor of Medicine, Bachelor of Surgery (MBBS) or its equivalent (MD, in the US, for example)[5].

### Stage 2: Identifying relevant studies

**Fig. 1:**
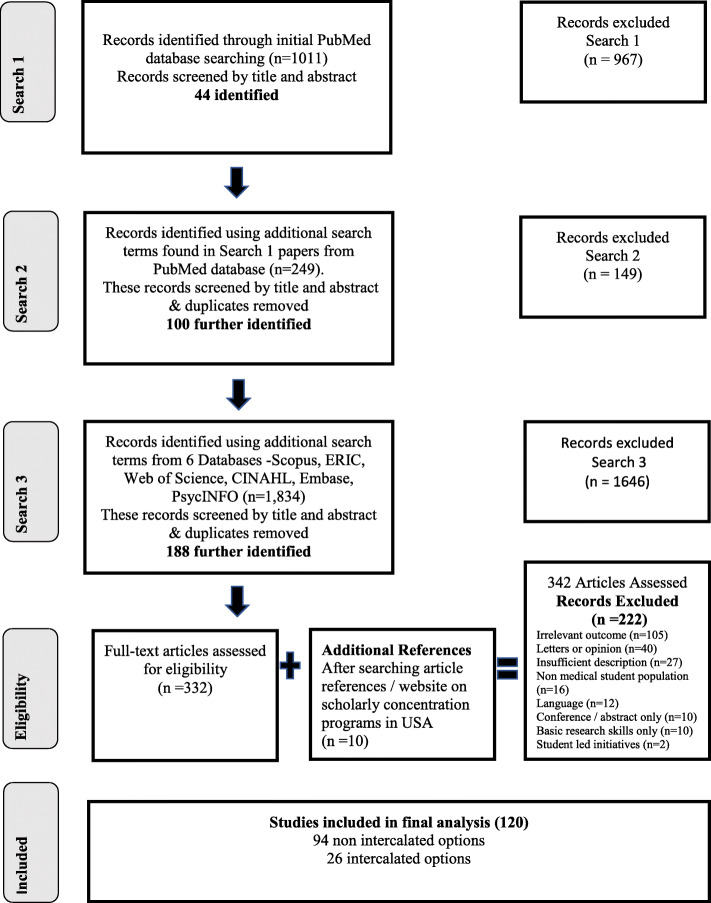
Identification of papers for inclusion in review using ‘PRISMA Extension for Scoping Reviews’ (PRISMA -SCR)

In the first instance, an initial search of PubMed was conducted with an analysis of text words contained in the title and abstract, and of the index terms used to describe the article. Four team members (CC / WC / JL / DS (librarian)) discussed the search terms. This initial search strategy identified 1011 studies of which 44 were selected to be read in full (see Fig. 1- PRISMA -SCR). Then, a second search using all identified keywords and index terms was conducted across all included databases: PubMed, Cumulative Index to Nursing and Allied Health Literature (CINAHL), PsycINFO, EMBASE, Scopus, Web of Science and ERIC (see Table 1).

**Table 1:** Refined search terms used

“Active research” OR “original research” OR “research project*” OR “research activit*” OR “research stud*” OR “special study option*” OR “special study component” OR “research elective*” OR “Doing research” OR “research skill*” OR “research competenc*” OR “translational medical research” OR “scholarly activity program*” OR theses OR “Research“[Mesh:NoExp] OR “Translational Medical Research“[Mesh] OR “Academic Dissertations” [Publication Type] OR “Academic Dissertations as Topic“[Mesh]
AND
“Medical student*” OR “student doctor*” OR “Students, Medical“[Mesh]
AND
“concurrent degree*” OR “combined degree*” OR “structured doctoral program*” OR “research degree*” OR “dual degree*” OR “intercalated degree*” OR “intercalated masters” OR “Medical curricul*” OR “Medical Degree Program*” OR “Medical Education” OR “Medical Program*” OR “Medical Degree” OR “MB Program*” OR “MB Degree Program*” OR “Bachelor of Medicine Degree Program*” OR “Bachelor of Medicine Program*” OR “MD Degree Program*” OR “Doctor of Medicine Degree Program*” OR “Doctor of Medicine Program*” OR “Pre-registration Medical Degree Program*” OR “Pre-registration Medical Program*” OR “Bachelor component of Bologna Cycle” OR “Education, Medical“[Mesh:NoExp] OR “Education, Medical, Undergraduate“[Mesh]

### Stage 3: Selection of studies

Consistent with the approach adopted by Arksey and O’Malley [20], our initial review of citations in PubMed indicated that the search strategy had identified 967 (1011-44) studies that were subsequently considered not relevant to the research question. As a result, the authors also had to devise some criteria post hoc in addition to the original inclusion and exclusion criteria in our protocol, that we could then apply to all the citations to determine their relevance. It was therefore an iterative process (see Table 2).

**Table 2:** Inclusion and exclusion criteria

	Inclusion Criteria	Exclusion Criteria
Population	• Medical students • Medical curriculum developers	• Postgraduate and/or non-medical students
Intervention	• Curriculum initiatives that describe elements of original or advanced research as per WFME 2015 definition • Curriculum initiatives that involve data collection by medical students	• Curriculum initiatives that pertain to WFME Basic Standards • Non-MB curriculum initiatives • Student Led Initiatives
Outcomes	• Academic performance • Demonstration of objective active research skills acquisition by the medical students • Feasibility issue discussion • Inspired students to do further research • Objective improvement in students research skills • Output of active research • Report curriculum developers’ motivations for active research curriculum initiatives • Report student motivations for doing research • Self-reported active research skills acquisition by the medical students • Sufficient description of the active research initiatives	• Insufficient description of the active research initiatives (i.e., not enough description of the research initiative for it to be reproduced by another medical school)
Study type	• Studies in the published literature in English / French / Spanish	• Studies in other languages apart from English / French / Spanish • Letters / Opinion papers

- A ‘PICO’ (population, intervention/exposure, comparison, outcome) analysis was carried out to facilitate identification of studies and develop the focus and scope of the review.
- The first author (CC) reviewed all 342 full text articles deemed eligible for the review. The second author (GM) reviewed 36 of the 342 full text articles. These 36 articles were articles in which CC was unsure if they met the inclusion criteria from the protocol. The authors could not reach agreement on whether seven articles should be included / excluded in the final review. Clarification was sought from the last author (WC) and agreement on which articles to include in the final review was reached.

After abstract and title screening, 332 papers were selected to be read in full. Having read the articles in full, ten additional articles were selected from reviewing the reference lists of selected studies and a collaborative website listing publications on ‘scholarly concentration programs’ in the USA [21]. (342). A total of 222 articles were ultimately excluded after analysis (see Fig. 1- PRISMA -SCR). The search process was guided by the PRISMA Extension for Scoping Reviews ( PRISMA -SCR) [22]. It maps out the number of records identified, included and excluded, and the reasons for exclusions. As this was a scoping review, the literature inclusion was broad. Ultimately 120 articles were selected for inclusion in this scoping review – 26 were related to intercalated degree options and 94 non intercalated degree options (see Fig. 1- PRISMA -SCR).

### Stage 4: Charting the data

The data was charted using Microsoft Excel and a ‘descriptive- analytical’ method within the narrative tradition was applied. The data extracted from each paper included:

- Author(s), year of publication, types of studies, study location.
- Main active research option described.
- Main outcome measure / other outcomes measures.
- Key results.
- Aim(s) of the study.

### Stage 5: Collating, summarising and reporting the results:

After subdividing into ‘Intercalated Degree’ and ‘Non intercalated Degree’ options, the year of publication, study location, aims for doing the study, main active research option and main outcome measure involved were collated for the included studies. With regards to study location, we included the 2020 World Bank definition of countries’ incomes as a proxy of medical school resources[23].

As these are a heterogeneous group of studies and outcomes, we have attempted to map the results into categories; one main outcome was included per study for this paper but some studies did have several outcomes which we have recorded. We chose the outcome measure that we felt the authors had highlighted as the main outcome measure. Thematic analysis was conducted for the key results for both the non -intercalated degree and intercalated options. The initial thematic analysis was completed by the first author (CC) using Braun and Clarke’s framework [24]. Steps 4 and 5 of the framework were then undertaken by the first, second and last author.

### Stage 6: Consultation

Healthcare education experts were consulted in relation to the scoping review findings in line with recommendations by Levac et al[19].

## Results

120 articles met the inclusion criteria and were selected for inclusion in the review, of which 94 related to non-intercalated degree options and 26 related to intercalated degree options. Results are listed for both the non-intercalated and the intercalated degree options below.

### Non-Intercalated Degree Options (*N* = 94)

The identified studies were published from 1976 to 2018. More of the selected studies were published in recent years with one being published in the 1970 s, five being published in the 1980 s, six in the 1990 s, 26 in the 2000 s and 56 since 2010. There was a wide geographical distribution of non-intercalated active research options. The majority were in the United States (40/94, 43 %) and the UK (7/94, 7 %). Several of the identified studies were from South America, the Middle East and Africa (see Table 3). In this table, we also included the World Bank definition of countries’ incomes as a proxy of medical school resources [23].

**Table 3:** Geographical distribution of non-intercalated studies / authors and World Bank definition of countries’ incomes as a proxy of medical school resources 2

Country	Frequency N = 94 n (%)	World Bank Income Economy Classification
United States	40 (43 %)	High Income
UK	7 (7 %)	High Income
Australia	5 (5 %)	High Income
The Netherlands / USA	3 (3 %)	High Income
Sweden	3 (3 %)	High Income
Canada	3 (3 %)	High Income
India	3 (3 %)	Lower Middle
Germany	3 (3 %)	High Income
Malaysia	3 (3 %)	Upper Middle
Turkey	2 (2 %)	Upper Middle
Norway	2 (2 %)	High Income
Other: Argentina, Australia / USA, Chile, Croatia, Germany / Croatia, Greece / UK, India / Malaysia, International, Ireland, Ireland / Malaysia, Japan, New Zealand, Pakistan, Portugal, Russia, Saudi Arabia, South Africa, Spain, UAE, West Indies	1 (1 %)	Varies

#### Types of studies

Most of the included studies were original research articles describing the active research option or the outcome of the active research option. This scoping review identified one randomised control trial in which some students undertook active research in pharmacology and compared various outcomes in this cohort to the other cohort who undertook the traditional pharmacology curriculum [25].

#### Aims of selected papers

There was heterogeneity in the aims of the selected studies. The top three aims were: to describe the programme experience (36/94; 38 %), to assess the impact of the research initiative on students (20/94; 21 %), and to describe the research output of the initiative (17/94; 18 %) (see Table 4).

**Table 4:** Aims for non-intercalated papers

Aims	Frequency (Total = 94) n (%)
To describe program experience	36 (38 %)
To assess impact / satisfaction of research initiative on students	21 (22 %)
To describe research output	17 (18 %)
To evaluate student attitudes to research	4 (4 %)
To examine the effect of undergraduate research on career	3 (3 %)
To stimulate student interest / increase recruitment to research	3 (3 %)
To try and increase trainees in a specialty by exposing them to research	2 (2 %)
To describe curricular content / guide curriculum design	2 (2 %)
To try and synthesise published studies on medical student research	1 (1 %)
To look at student motivations for research	1 (1 %)
To investigate research opportunities for medical students	1 (1 %)
To describe research options available	1 (1 %)
To discuss reasons for failure of research theses	1 (1 %)
To develop research capacity for all students	1 (1 %)

#### Main active research option described

The most described research option was elective research projects (a mixture of elective summer research projects and elective projects at other times of the year) followed by mandatory research projects at various stages of the medical curriculum. Other options included clinical audits incorporating research skills (see Table 5).

**Table 5:** Non-intercalated Active Research Options Frequency (94)

Research Option	Frequency (Total = 94) n (%)
*Mandatory Research Project(s)*	**40 (43 %)**
Mandatory research project (Longitudinal)	9 (10 %)
Mandatory research project (Various points in curriculum)	11 (12 %)
Mandatory research project (Final year)	5 (6 %)
Mandatory research project (Year 5)	1 (2 %)
Mandatory research project (Year 4)	3 (4 %)
Mandatory research project (Year 3)	4 (5 %)
Mandatory research project (Year 2)	3 (4 %)
Mandatory research project (Year 1)	3 (4 %)
Mandatory research project (Four weeks, not stated when in curriculum)	1 (2 %)
*Elective Research Project(s)*	**42 (45 %)**
Elective research project (Summer)	19 (21 %)
Elective research projects (Timing unspecified / varies)	18 (20 %)
Elective research project (Longitudinal)	3 (4 %)
Elective research project (One year)	2 (3 %)
*Mandatory and elective project(s)*	**7 (8 %)**
Mandatory and elective project(s)	7 (8 %)
*Unclear if mandatory or elective research project*	**1 (2 %)**
Unclear if mandatory or elective	1 (2 %)
*Audit project*	**3 (4 %)**
Mandatory audit project	1 (2 %)
Elective audit project	2 (3 %)
*List of options available in the ‘research projects section’*	**1 (2 %)**
Various options	1 (2 %)

#### Key findings reported of non-intercalated options

**Fig. 2:**
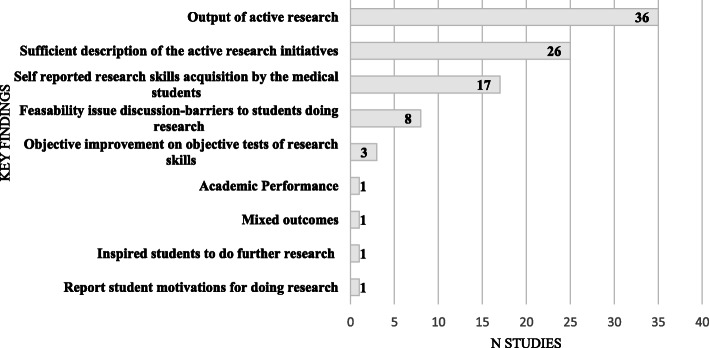
Key findings of non-intercalated active research options

The outcome measure most recorded was output of active research (publication rates, conference presentations) (36/94, 38 %). The next area reported was a quality measure of the primary studies which has been labelled as ‘sufficient description of the active research method’ (26/94, 28 %). We defined this as the ability of another medical school to reproduce the research initiative from the description given by the authors. (see Fig. 2).

### Intercalated Degree Options (*N* = 26)

The selected studies were published from 1985 to 2018. More of the selected studies were published in recent years with two being published in the 1980 s, one in the 1990 s, seven in the 2000 s and 16 since 2010. There was less geographical variation in the location of the intercalated degrees studies / authors with most being in the UK followed by the USA. As US medical programs are graduate entry (‘MD’), the included studies from the USA described intercalated masters (two) and PhDs (four). (see Table 6).

**Table 6:** Geographical distribution of intercalated studies / authors* and World Bank definition of countries’ incomes as a proxy of medical school resources

Country	Frequency (Total = 26)	World Bank Income Economy Classification
UK	13 (50 %)	High Income
USA	6 (23 %)	High Income
New Zealand	3 (12 %)	High Income
Australia	2 (7 %)	High Income
Chile * author	1 (4 %)	High Income
Germany	1 (4 %)	High Income

#### Main intercalated degree described

The most common intercalated degree described in the studies was an intercalated bachelor’s degree (14 papers), with intercalated BSc being most frequently described, followed by an intercalated PhD (six papers) (see Table 7).

**Table 7:** Intercalated Degree Frequency

Intercalated Degree Type	Frequency (Total = 26)
Intercalated Bachelor’s degree	14 (54 %)
Intercalated PhD	6 (23 %)
Intercalated Masters	4 (15 %)
Intercalated degrees various	2 (7 %)

#### Key findings reported

**Fig. 3:**
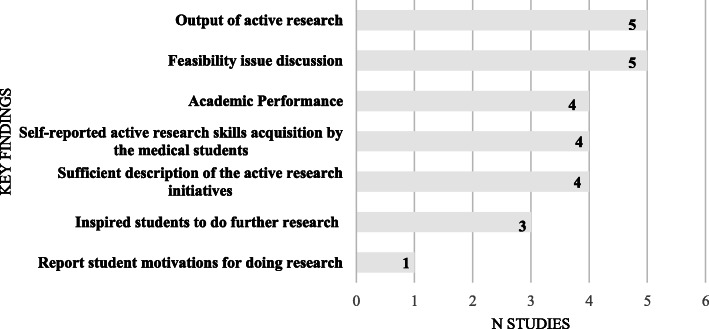
Key findings of intercalated degree options

Discussions on feasibility and output of the intercalated degrees (publication rates / conference presentations) were reported most (5/26, 19 % each) (see Fig. 3).

### Analysis of non-intercalated degree options key findings – outcomes, quality measure and themes.

#### Outcomes: (using Kirkpatrick criteria [26])

Thirty-six papers reported output of active research options (Kirkpatrick results criteria) of which 17 discussed both student publications and student presentations at conferences, 16 discussed student publications and three discussed student presentations at conferences.

Seventeen papers described medical students’ self-reported skills acquisition (Kirkpatrick reaction criteria). In nine of these papers, students reported an increase in their research skills or knowledge and in two of which students reported an increased awareness of their research identity / thinking as a scientist [27, 28].

Three studies carried out pre and post tests on the students’ research knowledge. There was an objective improvement in students’ research skills in all three studies (Kirkpatrick learning criteria). A different instrument was described in each of the three studies. [29–31]

#### Quality measure of primary studies: Sufficient description of active research option

Not all included papers had definite outcome measures recoded. 26 of them were included as they were rated to have had a sufficient description of the active research option. We defined this as the ability of another medical school to reproduce the research initiative from the description given by the authors. Papers were included if they included data such as the stage in the curriculum of the research option, duration of the option, the number of students taking part, research training given to students, discussed resources needed, student evaluation and assessment of the option(s). Not all papers in this group of papers had all these categories. For this group of papers, student-rated positive satisfaction with the research option was the predominant measure (12/25, 48 %). One study reported that students rated the experience lower than the teaching physicians involved [32].

#### Themes: Feasibility, mandatory versus elective research, time taken to do research and student research offices

Eight papers discussed feasibility issues or barriers to students doing research. A cross sectional study in 2016 looked at identifying underlying causes for failure of medical thesis projects and the constantly high drop-out rate in Germany from the supervisors’ perspective (response rate to the study was 29 %). The authors found that both thesis supervisors and medical students felt ill prepared for their roles in the process of a medical dissertation.[33]. Murdoch-Eaton et al’s 2015 mixed-method study across five medical schools found that students identified practical difficulties in preparation before the project commenced, including time commitment and applying for ethical committee approval, as potential impediments to choosing a project that might provide research [34].

There was a comparable number of papers reporting on mandatory or elective research projects in the 94 non intercalated studies, and neither approach found to be preferable. In a 2017 qualitative study comparing the impact of elective versus required medical school research experiences on career outcomes, researchers found that although completion of research at medical school correlated positively with current research involvement, a pre-existing passion for research was the greatest predictor for a career as a physician scientist (p = 0.008). Their study found that students who were primarily motivated by curricular requirement were less likely to pursue additional research opportunities. They reported that students who recalled positive medical school research experiences were more likely to pursue postgraduate research. The response rate to this study was low at 11.5 %. [35]

There was also a discussion about the issues in relation to mandatory theses in Germany [33, 36]. In the Diez paper, issues discussed by the authors included their thoughts on time pressures during medical school, how the dissertation was felt to be irrelevant to clinical practice and their perception that many projects were poorly designed. They discussed evidence from Germany that conducting research can delay graduation and may affect clinical skills because students working on projects attend fewer clinical teaching sessions and may not spend sufficient time preparing for examinations.

Dyrbye and colleagues found that prolonging the break from medical studies to do an intercalated degree / research for more than a year can have detrimental effects on student performances in clinical exams [37] and in a second paper found that shorter experience seemed to yield outcomes similar to longer experiences [38].

Several medical schools have developed ‘Student research offices’ in order to facilitate participation in extracurricular research [39, 40]. Tuncel et al. discussed ‘Student research clubs’ in a Turkish Medical School [41].

### Analysis of key findings of intercalated degree options – outcomes and ‘novel’ intercalated degree theme.

As previously discussed by Jones and Amgad in their systematic reviews [5, 12], the effect of doing an intercalated degree on medical student performance and careers was the most featured outcome measure.

#### Outcomes: Performance and student perspective on intercalating

Eight of the included 26 papers had publication rates, academic performance or careers in academic/research posts as a proxy measurement of the success of their respective intercalated degree programmes (Kirkpatrick results criteria). Five of the included 26 papers discussed the student perspective on doing intercalated degrees (Kirkpatrick reaction criteria). The top two motivations for planning to doing intercalated degrees in Agha et al’s study of 358 second-year medical students in London were career prospects and ‘a chance to gain publication’[42]. Park et al. found in their study in New Zealand of graduates who had completed an intercalated BSc from 1972–2005, that an interest in a career in research and academic medicine was the most commonly cited reason for undertaking an intercalated degree [43]. Stubbs et al. in their two centre cross sectional study in Bristol and Sheffield Medical Schools found that the participating students clearly valued the intercalated degrees and felt they gained a substantial advantage over their peers as well as skills helpful for their future careers [44].

Negative student perspectives on intercalated degrees included Agha et al’s findings that those who didn’t opt to intercalate cited financial (72 %) and time costs (44 %) and lack of interest in doing research (48 %) as the main reasons given [42]. Nicholson et al. reported in their study in Aberdeen that the most common reason students opted not to intercalate was because they did not want to have another year of study (69.6 %) or incur more debt (51.9 %)[45].

Eighty per cent of respondents in the study by Park et al. encountered some problems during the intercalated year, with the most common reported being loss of contact with friends in the medical course [43]. There was a small number involved in this study (30 / 50 responded).

#### Theme: More ‘novel’ intercalated degrees than traditional intercalated BSc

An intercalated BSc [46, 47] and Masters [48] in primary care were discussed. Jones et al. felt that intercalated degrees could be aimed at a broader group of students than those wanting teaching hospital consultant posts or laboratory based science careers. Elwood et al. discussed the community health option of a mandatory BSc in Nottingham as an alternative to the more traditional BSc [49]. Muir et al. described a medical education intercalated BMSc in Dundee in which students enhanced their learning through collaboration and opportunities gaining a better appreciation of the roles and responsibilities of staff roles and academic clinicians [50]. Pearson et al. described an intercalated BSc which involved students doing field research in foreign, lower income countries [51]. Stellman et al. discussed a one-year Master’s of Public Health that students took between their third and fourth years in New York [52]. Menger et al. discussed an intercalated MB / PhD programme in Germany in which students perform a PhD thesis on a surgery-related research topic in their 6th year as a culmination of a research program from the third year [53]. The aim of this programme is to increase the number of surgeon scientists.

## Discussion

### Key findings

This scoping review has mapped the various options available to medical schools to enhance research experiences for medical students which range from mandatory intercalated degrees to elective research projects.

There was heterogeneity in the context and methodologies of the included papers, and few involved experimental design. The main outcomes reported for the non-intercalated degree options were student research output and self-reported research skills acquisition by medical students. Miscellaneous themes pertained to feasibility issues and whether opportunities to ‘do research’ should be mandatory or optional for medical students. For the intercalated degree options, the three main areas discussed were descriptions of more ‘novel’ intercalated degree options than the traditional BSc, student perspectives on intercalating and the effect of intercalating on medical student performance and careers.

### How findings relate to other literature

Any changes to a medical curriculum need to be considered in light of rapidly evolving health system needs, structure and performance. The past number of years have seen a wave of calls for curriculum reform, new accreditation standards and regulatory requirements [54]. The century old paradigm of “2 + 2” basic science and clinical clerkship and even discipline-based education have been challenged.

Studies to date that have synthesised research opportunities for medical students [5, 12] have focused on medical student exam performance and career progression which did show an overall positive effect. Amgad et al. discussed the fact that students are deterred by practical difficulties, including the lack of opportunities and funding. The concerns of Cheung, Parsonnet and Dyebye in relation to medical student research were discussed earlier in this paper and include cost, mentor availability, student motivations for doing research, potential detrimental effects of time out from clinical studies and quality of medical student publications [15, 16, 37]. Transferable skills such as communication, teamwork, problem solving and networking that students can develop when students do research are key to the development of future doctors but are harder to measure [55]; however, these are also valuable attributes for further investigation.

More work needs to be done to ascertain whether opportunities to ‘do research’ should be mandatory or optional in a medical curriculum, as based on current research, flexibility is probably appropriate. Concerns have been raised about the detrimental effects mandatory theses may have on medical students [33, 36]. An important area to consider regarding flexibility includes the individual medical school’s resources. Using World Bank definitions of countries’ incomes, we noted that there were fewer selected studies from medical schools in lower-middle- and low-income countries, where student involvement in original research may not be the priority to address local healthcare needs in these curricula. However, a paper by Salloum et al. found that there appeared to be the same appetite in medical students to do research from high and lower income countries [56].

In some higher income countries with more resources, an emphasis has been put on medical student research experience by governments to try and increase the number of physician- scientists. For example, in the US, the National Institutes of Health (NIH) sponsored Medical Student Research Fellowship Programmes [6]. An example from Europe is the Norwegian Medical Student Research Programme, which is a Norwegian government led initiative initiated in 2002 in response to a falling number of doctors recruited to medical research in Norway in the 1990 s [57]. The authors reported that this Medical Student Research Programme had led to an increase in the recruitment of graduated physicians to medical research in Norway.

Equally, the profile of medical course student populations (e.g., undergraduate entry versus graduate entry medical students) has a bearing on a medical school’s curriculum. Accelerated, graduate-entry medicine courses tend to have particularly full curricula and these students may have prior research experience in a previous degree [58]. Consideration also has to be taken into account of the effect of time allocated to research and potentially detrimental performances in clinical examinations, as mandatory research may therefore not be suitable for all students [37]. Medical schools can also nurture student involvement in research by supporting student led initiatives such as student led research conferences, research clubs and student medical journals [59].

### Methodological considerations

This scoping review approach has several limitations. Scoping reviews do not formally evaluate the quality of evidence and often gather information from a wide range of study designs and methods [60]. By design, the number of studies included in this review process was thus sizeable, and the results could not be synthesised into distinct outcomes. Only one main outcome was recorded per study for summarising for this paper, this could have introduced bias. As with other scoping reviews, the authors have not provided a synthesised result or answer to a specific question, but rather provided an overview of the available literature on ‘doing research’ options available to medical schools.

The heterogeneity of included papers makes it difficult to draw conclusions on our study aim to explore options available to medical schools but heterogeneity as a review outcome is common to other medical education literature reviews [61, 62]. The published studies encountered in this scoping review reported more positive than negative outcomes in relation to active student research activities. The authors are aware that this may be affected by publication bias, resource availability and to the difficulty of putting a net value on the overall benefit of active research involvement of medical students, given the heterogeneity of the studies and outcome measures published from research output to students’ self-reported research self-efficacy Kappa scores were not carried out.

### Implications for research, education, practice and policy

Further research is needed to ascertain if ‘doing research’ options should be more emphasised in medical curricula. By describing the research experiences available, we have provided a possible starting block to evaluate the effectiveness of the research experiences offered to medical students. Evaluating the effectiveness of the research options may help shape medical education policy on research and medical workforce planning in the training of physician scientists. Consideration could be given to using Theory Based Evaluation (TBE) to measure ‘success’ of research initiatives for students [63, 64]. For now, flexibility is probably appropriate depending on a medical school’s resources, curriculum, educational culture and population needs.

## Conclusions

We have described educational activity that involved actively conducting research (‘doing research’) for students on pre-registration medical degree programmes with elective research projects being the most common described in the selected studies. The studies included in this review reported more positive than negative outcomes in relation to active student research activities. Positive findings included research output, research self-efficacy and career development. At the same time cost, mentor availability, student motivations for doing research, potential detrimental effects of time out from clinical studies and quality of medical student publications need to be considered.

## Data Availability

The datasets used and/or analysed during the current study are available from the corresponding author on reasonable request.

## Footnotes

2 For the 2020 fiscal year, low-income economies are defined as those with a GNI per capita, calculated using the World Bank Atlas method, of $1,025 or less in 2018; lower middle-income economies are those with a GNI per capita between $1,026 and $3,995; upper middle-income economies are those with a GNI per capita between $3,996 and $12,375; high-income economies are those with a GNI per capita of $12,376 or more

## References

[1] Sung NS, Crowley WF, Genel M, Salber P, Sandy L, Sherwood LM (2003). Central challenges facing the national clinical research enterprise. Jama.

[2] Cooke M, Irby DM, Sullivan W, Ludmerer KM (2006). American medical education 100 years after the Flexner report. New England journal of medicine.

[3] Jain MK, Cheung VG, Utz PJ, Kobilka BK, Yamada T, Lefkowitz R (2019). Saving the Endangered Physician-Scientist — A Plan for Accelerating Medical Breakthroughs. New England Journal of Medicine.

[4] Reinders JJ, Kropmans TJB, Cohen Schotanus J. Extracurricular research experience of medical students and their scientific output after graduation. Med Educ. 2005;39:237. 10.1111/j.1365-2929.2004.02078.x 15679693

[5] Amgad M, Man Kin Tsui M, Liptrott SJ, Shash E (2015). Medical Student Research: An Integrated Mixed-Methods Systematic Review and Meta-Analysis. PLoS One.

[6] Solomon SS, Tom SC, Pichert J, Wasserman D, Powers AC (2003). Impact of medical student research in the development of physician-scientists. J Investig Med.

[7] Stringer MD, Ahmadi O. Famous discoveries by medical students. ANZ J Surg. 2009;79:901–8. 10.1111/j.1445-2197.2009.05142.x 20002992

[8] WFME. World federation for medical education Basic Medical Education WFME Global Standards for Quality Improvement The 2015 Revision. 2015.

[9] Laidlaw A, Aiton J, Struthers J, Guild S (2012). Developing research skills in medical students: AMEE Guide No. 69. Medical Teacher.

[10] GMC. Outcomes for graduates document 2018 [Available from: https://www.gmc-uk.org/education/standards-guidance-and-curricula/standards-and-outcomes/outcomes-for-graduates/outcomes-for-graduates/outcomes-3---professional-knowledge#clinical-research-and-scholarship.

[11] Marz R, Dekker FW, Van Schravendijk C, O’Flynn S, Ross MT (2013). Tuning research competences for Bologna three cycles in medicine: report of a MEDINE2 European consensus survey. Perspectives on medical education.

[12] Jones M, Singh S. Impact of an intercalated BSc on medical student performance and careers: A BEME systematic review: BEME Guide No. 28. Medical Teacher. 2013. 10.3109/0142159X.2013.806983 23962229

[13] Alamri Y (2018). Dual Degrees in Medicine: Options for Medical Students. J Cancer Educ.

[14] Simunovic F (2008). Is there a place for medical students in research laboratories? A student’s perspective. Med Teach.

[15] Parsonnet J, Gruppuso PA, Kanter SL, Boninger M (2010). Required vs. elective research and in-depth scholarship programs in the medical student curriculum. Academic Medicine.

[16] Cheung BM (2018). Medical student research: is it necessary and beneficial?. Postgraduate medical journal.

[17] Pathipati AS, Taleghani N. Research in medical school: a survey evaluating why medical students take research years. Cureus. 2016;8(8):e741. 10.7759/cureus.741. 10.7759/cureus.741 PMC5026499 27672532

[18] Pham MT, Rajić A, Greig JD, Sargeant JM, Papadopoulos A, McEwen SA (2014). A scoping review of scoping reviews: advancing the approach and enhancing the consistency. Research synthesis methods.

[19] Levac D, Colquhoun H, O’Brien KK. Scoping studies: advancing the methodology. Implementation science. 2010;5(1):1–9. 10.1186/1748-5908-5-69 PMC2954944 20854677

[20] Arksey H, O’Malley L (2005). Scoping studies: towards a methodological framework. International journal of social research methodology.

[21] The University of Chicago Pritzker School of Medicine. Publications and Presentations of Scholarly Concentration Collaborative [Available from: http://time.uchicago.edu/sccollaborative/publications-and-presentations/.

[22] Tricco AC, Lillie E, Zarin W, O’Brien KK, Colquhoun H, Levac D (2018). PRISMA extension for scoping reviews (PRISMA-ScR): checklist and explanation. Annals of internal medicine.

[23] World Bank. World Bank Country and Lending Groups 2020 [Available from: https://datahelpdesk.worldbank.org/knowledgebase/articles/906519-world-bank-country-and-lending-groups#:~:text=For%20the%20current%202020 %20fiscal,those%20with%20a%20GNI%20per.

[24] Braun V, Clarke V (2006). Using thematic analysis in psychology. Qualitative research in psychology.

[25] Tamayo G, Santibañez M, Meana JJ (2005). Evaluation of a pharmacology educational activity based on a research project: A randomized, controlled and blind analysis of medical students’ perceptions. Medical Teacher.

[26] Kirkpatrick DL, Craig R. Evaluation of training. Evaluation of short-term training in rehabilitation. 1970:35.

[27] Möller R, Shoshan M, Heikkilä K (2015). What is the reward? Medical students’ learning and personal development during a research project course. Medical Education Online.

[28] Ahmad A, Bahri Yusoff MS, Zahiruddin Wan Mohammad WM, Mat Nor MZ (2018). Nurturing professional identity through a community based education program: medical students experience. Journal of Taibah University Medical Sciences.

[29] Griswold K, Silverstein D, Lenkei E, Fiedler R (1991). Research skills for medical students: a summer assistantship in family medicine. Fam Med.

[30] Knight SE, Van Wyk JM, Mahomed S (2016). Teaching research: a programme to develop research capacity in undergraduate medical students at the University of KwaZulu-Natal, South Africa. BMC Med Educ.

[31] Vereijken MW, van der Rijst RM, van Driel JH, Dekker FW (2018). Student learning outcomes, perceptions and beliefs in the context of strengthening research integration into the first year of medical school. Advances in Health Sciences Education.

[32] Moßhammer D, Mörike K, Lorenz G, Joos S (2016). Research tasks as part of the general practice clerkship in undergraduate medical education - A pilot project on feasibility and acceptance. Education for Primary Care.

[33] Can E, Richter F, Valchanova R, Dewey M. Supervisors’ perspective on medical thesis projects and dropout rates: Survey among thesis supervisors at a large German university hospital. BMJ Open. 2016;6(10). 10.1136/bmjopen-2016-012726 PMC5073490 27742631

[34] Murdoch-Eaton D, Drewery S, Elton S, Emmerson C, Marshall M, Smith JA (2010). What Do Medical Students Understand By Research And Research Skills? Identifying Research Opportunities Within Undergraduate Projects. Medical Teacher.

[35] Weaver AN, McCaw TR, Fifolt M, Hites L, Lorenz RG (2017). Impact of elective versus required medical school research experiences on career outcomes. J Investig Med.

[36] Diez C, Arkenau C, Meyer-Wentrup F. The German medical dissertation- time to change? Acad Med. 2000;75. 10.1097/00001888-200008000-00024 10965870

[37] Dyrbye LN, Thomas MR, Natt N, Rohren CH (2007). Prolonged delays for research training in medical school are associated with poorer subsequent clinical knowledge. Journal of General Internal Medicine.

[38] Dyrbye LN, Davidson LW, Cook DA (2008). Publications and presentations resulting from required research by students at Mayo Medical School, 1976–2003. Acad Med.

[39] Zier K, Friedman E, Smith L (2006). Supportive programs increase medical students’ research interest and productivity. J Investig Med.

[40] Nikkar-Esfahani A, Jamjoom AAB, Fitzgerald JEF (2012). Extracurricular participation in research and audit by medical students: Opportunities, obstacles, motivation and outcomes. Medical Teacher.

[41] Tuncel H, Korpinar MA (2015). Voluntary student research groups in medical education: Teaching teamwork. FEBS Journal.

[42] Agha R, Howell S (2005). Intercalated BSc degrees–why do students do them?. The Clinical Teacher.

[43] Park SJK, Liang MMS, Sherwin T, McGhee CNJ (2010). Completing an intercalated research degree during medical undergraduate training: Barriers, benefits and postgraduate career profiles. New Zealand Medical Journal.

[44] Stubbs TA, Lightman EG, Mathieson P. Is it intelligent to intercalate? A two centre Cross-sectional study exploring the value of intercalated degrees, and the possible effects of the recent tuition fee rise in England. BMJ Open. 2013;3(1):e002193. 10.1136/bmjopen-2012-002193. 10.1136/bmjopen-2012-002193 PMC3563132 23355672

[45] Nicholson JA, Cleland J, Lemon J, Galley HF. Why medical students choose not to carry out an intercalated BSc: A questionnaire study. BMC Med Educ. 2010;10:25. Published 2010 Mar 23. 10.1186/1472-6920-10-25. 10.1186/1472-6920-10-25 PMC2850914 20331878

[46] Jones M, Lloyd M, Meakin R (2001). An intercalated BSc in Primary Health Care - An outline of a new course. Medical Teacher.

[47] Jones M, Singh S, Lloyd M. “It isn’t just consultants that need a BSc”: Student experiences of an intercalated BSc in Primary Health Care. Medical Teacher. 2005;27(2):164–8. 10.1080/01421590400019567 16019339

[48] Creavin ST, Mallen CD, Hays RB (2010). An intercalated research Masters in primary care: a pilot programme. Education for Primary Care.

[49] Elwood JM, Pearson JC, Madeley RJ, Logan RF, Beaver MW, Gillies PA (1986). Research in epidemiology and community health in the medical curriculum: students’ opinions of the Nottingham experience. J Epidemiol Community Health.

[50] Muir F, Law S (2014). Students’ perceptions and experiences of a new “Teaching in Medicine” BMSc intercalated degree programme. Med Teach.

[51] Pearson S, Parr J, Ullah Z, Omar M (2014). Supporting medical students to do international field research: A case study. Innovations in Education and Teaching International.

[52] Stellman JM, Cohen S, Rosenfield A (2008). Evaluation of a one-year masters of public health program for medical students between their third and fourth years. Academic Medicine.

[53] Menger MD, Schilling MK, Schäfers HJ, Pohlemann T, Laschke MW (2012). How to ensure the survival of the surgeon-scientist? the Homburg Program. Langenbeck’s Archives of Surgery.

[54] Thomas PA, Kern DE, Hughes MT, Chen BY. Curriculum development for medical education: a six-step approach: JHU Press; 2016. 10.1097/ACM.0000000000002580 30681454

[55] Burgoyne LN, O’Flynn S, Boylan GB. Undergraduate medical research: the student perspective. Med Educ Online. 2010;15. 10.3402/meo.v15i0.5212. 10.3402/meo.v15i0.5212 PMC2939395 20844608

[56] Salloum RH, Nazha B, Zgheib NK. Interest and involvement in research during medical school: a global comparison of students at high-and low-income universities. Med Sci Educ. 2014;24:65–73.

[57] Hunskaar S, Breivik J, Siebke M, Tommeras K, Figenschau K, Hansen J. Evaluation of the medical student research programme in Norwegian medical schools. A survey of students and supervisors. BMC Med Educ. 2009;9:43. 10.1186/1472-6920-9-43. 10.1186/1472-6920-9-43 PMC2720957 19602226

[58] Jones R, Higgs R, De Angelis C, Prideaux D (2001). Changing face of medical curricula. The Lancet.

[59] Funston G (2015). The promotion of academic medicine through student-led initiatives. International journal of medical education.

[60] Sucharew H, Macaluso M (2019). Methods for Research Evidence Synthesis: The Scoping Review Approach. Journal of hospital medicine.

[61] Reed D, Price EG, Windish DM, Wright SM, Gozu A, Hsu EB (2005). Challenges in systematic reviews of educational intervention studies. Annals of Internal Medicine.

[62] Prideaux D, Bligh J (2002). Research in medical education: asking the right questions. Medical Education.

[63] Bierer SB, Chen HC (2010). How to measure success: the impact of scholarly concentrations on students—a literature review. Academic Medicine.

[64] Bierer SB, Prayson RA, Dannefer EF (2015). Association of research self-efficacy with medical student career interests, specialization, and scholarship: a case study. Advances in Health Sciences Education.

